# The role of the nucleus in the control of mitochondrial precursor proteins in yeast

**DOI:** 10.1002/pro.70622

**Published:** 2026-05-18

**Authors:** Kira Ritzenhofen, Stefano Rossi, Axel Mogk, Fabian den Brave

**Affiliations:** ^1^ Faculty of Medicine, Institute of Biochemistry and Molecular Biology University of Bonn Bonn Germany; ^2^ Center for Molecular Biology of Heidelberg University (ZMBH) DKFZ‐ZMBH Alliance Heidelberg Germany

**Keywords:** chaperones, mitochondria, nucleus, protein quality control, protein sorting, stress response, ubiquitin‐proteasome system

## Abstract

Mitochondria are essential organelles of eukaryotic cells, with vital roles in energy production, biosynthesis of macromolecules, and intracellular signaling. Their function depends on a complex proteome with proteins targeted to different mitochondrial sub‐compartments. Synthesis of precursors of mitochondrial proteins (mitoPREs) mostly occurs in the cytosol followed by post‐translational import. Delay or block of mitochondrial import leads to mitoPRE accumulation in the cytosol, where they interact with cytosolic protein quality control (PQC) factors and might get re‐routed to other cellular organelles, including the nucleus. Recent research implies the nucleus as a central hub in cellular PQC. Here, not only nuclear but also proteins from other organelles, including mitochondria or the cytosol, are handled by intra‐nuclear PQC factors. In addition, the nucleus controls the expression of mitochondrial proteins and PQC components involved in handling mitoPREs and surveilling the integrity of mitochondrial import channels. In this review, we discuss recent insights from yeast on the dual function of the nucleus in controlling the biogenesis of mitoPREs and as a compartment for quality control of non‐imported mitoPREs. We additionally describe how mitochondrial dysfunction and defects in mitochondrial import trigger compensatory stress responses inside the nucleus. Here, nuclear targeting of non‐imported mitoPREs may serve as a direct signal to adjust stress response pathways to the status of mitochondrial import.

## 1 INTRODUCTION

Compartmentalization with distinct organelles is a hallmark of eukaryotic cells requiring a sophisticated network of inter‐organellar interactions and communication to ensure cellular functionality. Each organelle requires a dedicated set of proteins, which are, with the exception of a few mitochondrial proteins, encoded in the nuclear DNA. Following transcription, these proteins are synthesized on cytosolic ribosomes and subsequently further sorted, whereby two‐thirds of the cell's proteins have to be transported into or across at least one membrane (Song & Becker, 2022). Within eukaryotic cells, mitochondria are central for energy production, metabolism and signaling. The mitochondrial proteome comprises about 1000 (in baker's yeast) to 1500 (in humans) proteins, of which 99% are encoded in the nuclear genome (Morgenstern et al., 2017; Morgenstern et al., 2021; Rath et al., 2021). Depending on the metabolic state, mitochondria can occupy up to 40% of the cellular volume, with the inner mitochondrial membrane as the protein richest cellular membrane, comprising proteins of the respiratory chain and a large set of metabolite carriers (Di Bartolomeo et al., 2020). Mitochondrial import of nuclear encoded proteins can occur in a co‐translational manner, however, the majority of these proteins are imported post‐translationally (Bykov et al., 2020; Song & Becker, 2022). Thus, following synthesis, mitochondrial precursor proteins (mitoPREs) are transiently present in the cytosol, where they are handled by the cytosolic protein quality control (PQC) machinery. Consequently, accumulation of mitoPREs in the cytosol can sequester cytosolic factors and thereby impair cellular protein homeostasis (Nowicka et al., 2021; Pfanner et al., 2025; Schlagowski et al., 2021; Wang & Chen, 2015). Thus, nuclear transcription of mitochondrial and PQC factors has to be coordinated and adapted to the mitochondrial import capacity (Boos et al., 2019; Wang & Chen, 2015; Weidberg & Amon, 2018; Wrobel et al., 2015; Yuan et al., 2026). This is achieved through extensive crosstalk between the nucleus and mitochondria to adapt transcriptional programs for mitochondrial biogenesis.

## 2 MITOCHONDRIAL TARGETING AND IMPORT

Following synthesis on cytosolic ribosomes mitoPREs are guided to mitochondria, where they are sorted into the different mitochondrial sub‐compartments. For this, the vast majority of mitoPREs first has to pass the outer membrane through the translocase of the outer membrane (TOM) complex. Targeting to mitochondria is guided by N‐terminal or internal mitochondrial targeting sequences (MTS). About 60% of mitoPREs contain an N‐terminal presequence which is cleaved off after import by intra‐mitochondrial proteases (Vögtle et al., 2009). Receptors at the TOM‐complex recognize the targeting signals, thereby guiding import. Tom20 and Tom22 are the receptors for proteins with N‐terminal presequences, while internal targeting signals are recognized by Tom70 (Backes et al., 2018; Yamamoto et al., 2009; Yamano et al., 2008). In order to pass through the central pore of the TOM complex, mitoPREs need to be maintained in a largely unfolded state (Wiedemann et al., 2001; Zhou et al., 2023). In the cytosol, molecular chaperones of the Hsp70 and Hsp90 family and their dedicated co‐chaperones play dual roles in preventing premature precursor folding and guide proteins to the mitochondrial surface (Bykov et al., 2020). Here, Hsp70 and Hsp90 can directly interact with the tetratricopeptide (TPR) domain of the TOM subunit Tom70 (Backes et al., 2018; Young et al., 2003). Specificity of Hsp70 is controlled through J‐domain proteins (JDPs), which promote Hsp70 substrate interaction and stimulate Hsp70's ATP hydrolysis. In mitochondrial targeting the general, highly abundant JDPs Ydj1 and Sis1 bind to mitoPREs and promote their Hsp70 interaction (Drwesh et al., 2022; Jores et al., 2018). In addition, more specialized JDPs like Xdj1 and Djp1 bind to different receptors of the TOM complex, Tom22 and Tom70, respectively (Opaliński et al., 2018). With the exception of most outer membrane proteins with α‐helical transmembrane segments, all mitochondrial proteins first pass the outer membrane via the TOM complex. Subsequently, proteins are then further sorted into the outer or inner membrane, intermembrane space or mitochondrial matrix by dedicated import machineries (Busch et al., 2023). Integration of *β*‐barrel proteins into the outer membrane is mediated by the sorting and assembly machinery (Paschen et al., 2003; Wiedemann et al., 2003), while intermembrane space proteins are sorted via the mitochondrial intermembrane space assembly pathway (Chacinska et al., 2004; Mesecke et al., 2005). Presequence containing proteins are sorted into or across the inner membrane by the presequence translocase TIM23 complex (Geissler et al., 2002; Truscott et al., 2001; Yamamoto et al., 2002), which is assisted by the presequence translocase associated motor (PAM) complex (D'Silva et al., 2005; Li et al., 2004; Mokranjac et al., 2006; van der Laan et al., 2005). Integration of multi‐spanning proteins such as metabolite carrier into the inner membrane is guided by the carrier translocase TIM22 complex (Rehling et al., 2003). Import into or across the inner membrane requires the membrane potential across the inner membrane, which is generated by the respiratory chain.

Inefficient import can have multiple causes, which lead to an accumulation of mitoPREs in the cytosol (Pfanner et al., 2025). For instance, defects in the respiratory chain result in a reduced import at the inner membrane due to a lowered membrane potential. Moreover, premature folding prevents import and partial folding of a mitoPRE can even result in precursor arrest during translocation, thereby blocking the import channel. Moreover, mutations in mitoPREs, as shown for pathogenic variants of the mitochondrial ATP/ADP carrier, can arrest at the TOM complex (Coyne et al., 2023). Likewise, several aggregation‐prone proteins linked to neurodegenerative diseases in humans such as *α*‐synuclein, Tau, or amyloid‐precursor protein have been shown to accumulate at the mitochondrial TOM complex, thereby impairing mitochondrial import (Devi et al., 2006; Di Maio et al., 2016; Needs et al., 2023).

**FIGURE 1:**
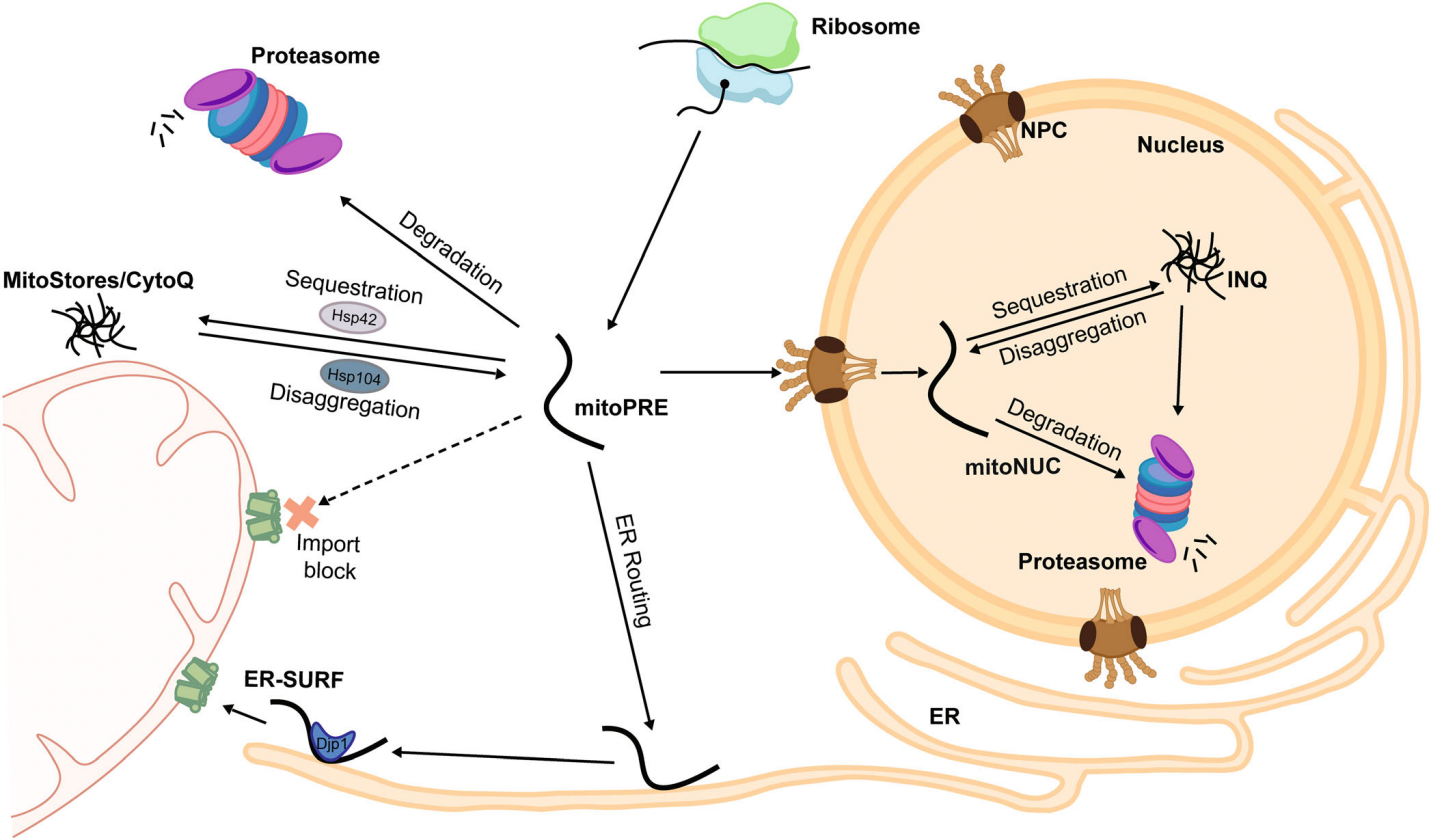
General protein quality control strategies upon mitoPRE accumulation in the cytosol. Mitochondrial precursor proteins (mitoPREs) are synthesized in the cytosol and targeted to mitochondria mostly in a post‐translational manner. Block or overload of mitochondrial import leads to the accumulation of mitoPREs, posing a threat to cellular proteostasis and demanding for their handling by protein quality control (PQC) activities. mitoPREs can be (i) targeted to proteasomal degradation or (ii) sequestered at specific inclusions (CytoQ/Mitostores) by the sHsp Hsp42. Sequestered mitoPREs can be rescued by protein disaggregation involving the Hsp104 disaggregase. mitoPREs can also be targeted to the nucleus, where they are subjected to similar PQC activities. Alternatively, mitoPREs are routed to the endoplasmic reticulum (ER) for later retargeting to mitochondria via the ER‐SURF pathway mediated by the J‐domain protein Djp1. CytoQ, cytosolic quality control compartment; INQ, intranuclear quality control compartment; mitoNUC, nuclear‐associated mitoprotein degradation.

The arrest of mitoPREs at TOMs is counteracted by multiple quality control mechanisms that extract precursors from the TOM complex and mediate their degradation by the cytosolic ubiquitin‐proteasome system. Here, proteins are first extracted from the TOM complex by the conserved AAA‐ATPases Cdc48 and Msp1 and subsequently targeted for proteasomal degradation (Basch et al., 2020; Mårtensson et al., 2019; Schulte et al., 2023; Weidberg & Amon, 2018). However, severe mitochondrial damage, impaired cytosolic proteostasis, or ongoing clogging of the TOM complex will result in proteotoxic accumulation of mitoPREs in the cytosol. Thus, cells have developed a multitude of strategies to deal with non‐imported mitoPREs and limit their burden on cellular proteostasis (Figure 1).

## 3 FATE OF NON‐IMPORTED PRECURSOR PROTEINS

Most mitochondrial proteins will not fold properly in the cytosol due to the presence of hydrophobic transmembrane domains, the lack of dedicated assembly partners, the presence of presequences or the requirement of the oxidative environment of the intermembrane space for correct folding. Thus, mitoPREs in the cytosol are often metastable and expose hydrophobic residues, potentially interacting with other proteins or membranes, disturbing cellular proteostasis which may eventually cause cell death (Nowicka et al., 2021; Schlagowski et al., 2021; Wang & Chen, 2015). In addition, cellular proteostasis is further disturbed by the sequestration of cytosolic quality control factors binding to mitoPREs. Moreover, some mistargeted mitochondrial enzymes may become active within the cytosol, affecting cellular metabolism (Nishio et al., 2023). The main strategies to deal with non‐imported mitoPREs are degradation and spatial sequestration, which are often interconnected (Figure 1). In general, non‐imported mitoPREs are subjected to proteasomal degradation (Bragoszewski et al., 2013; Kowalski et al., 2018). This involves the modification of mitoPREs with ubiquitin, which in turn inhibits import, as ubiquitylated proteins cannot pass through the TOM complex (Bragoszewski et al., 2013).

Upon import inhibition or heat shock, mitoPREs in the cytosol can be ubiquitylated by the E3 ligase Rsp5, which directly targets substrates containing PPxY motifs or indirectly via binding to Tom70 or the abundant cytosolic JDP Ydj1 (Fang et al., 2014; Schulte et al., 2023). However, ubiquitylation of precursor proteins at the TOM complex can be counteracted by the TOM‐bound deubiquitylating enzyme Ubp16, allowing import to proceed (Schulte et al., 2023). In addition to ubiquitylation, mitoPREs in the cytosol are modified with the ubiquitin‐like protein SUMO (Paasch et al., 2018). SUMOylation of mitoPREs is triggered by impaired mitochondrial import or defects in cytosolic proteostasis, suggesting a link to quality control. The consequence of mitoPRE SUMOylation is still unclear.

Although direct turnover by the proteasome is conceivably the most direct way to deal with non‐imported mitoPREs, research efforts of recent years have uncovered a variety of alternative strategies. A global analysis of GFP‐tagged mitoPREs upon import impairment by membrane potential dissipation has revealed targeting to the cytosol, the mitochondrial surface, the endoplasmic reticulum (ER) and into the nucleus (Shakya et al., 2021). Re‐localization to the ER has mainly been observed for hydrophobic inner membrane proteins such as metabolite carriers or the inner membrane insertase Oxa1 (Hansen et al., 2018; Shakya et al., 2021; Xiao et al., 2021). For some mitochondrial carriers it was described that ER targeting depends on components of the guided entry of tail‐anchored proteins (GET) pathway, which is the canonical pathway for ER targeting of tail‐anchored proteins (Xiao et al., 2021). An integral ATPase at the ER called Spf1 has been shown to extract mislocalized mitoPREs from the ER (McKenna et al., 2020; Qin et al., 2020). In addition, the integral ER protein Ema19 can mediate the degradation of ER‐localized mitoPREs (Laborenz et al., 2021). For several mitoPREs the ER‐associated degradation machinery supports their targeting for proteasomal degradation, depending on the ER membrane bound ubiquitin‐ligase Doa10 (Dederer et al., 2019). Importantly, in mitoPRE sorting the ER surface also supports re‐targeting to mitochondria via a pathway termed ER‐SURF, where the JDP Djp1 mediates the transport of mitoPREs from the ER surface to mitochondria via Tom70 of the TOM complex (Figure 1) (Hansen et al., 2018; Koch et al., 2024). Thus, ER accumulation of mitoPREs upon impaired import might also be caused by an interruption of this targeting route. Deletion of Ema19 increases the re‐targeting of mitoPREs to mitochondria, suggesting that proteasomal and ER‐SURF activities are tightly balanced (Laborenz et al., 2021). An increased accumulation of mitoPREs also serves as an explanation for the observed induction of the unfolded protein response (UPR) upon impairment of mitochondrial import (Knöringer et al., 2023).

**FIGURE 2:**
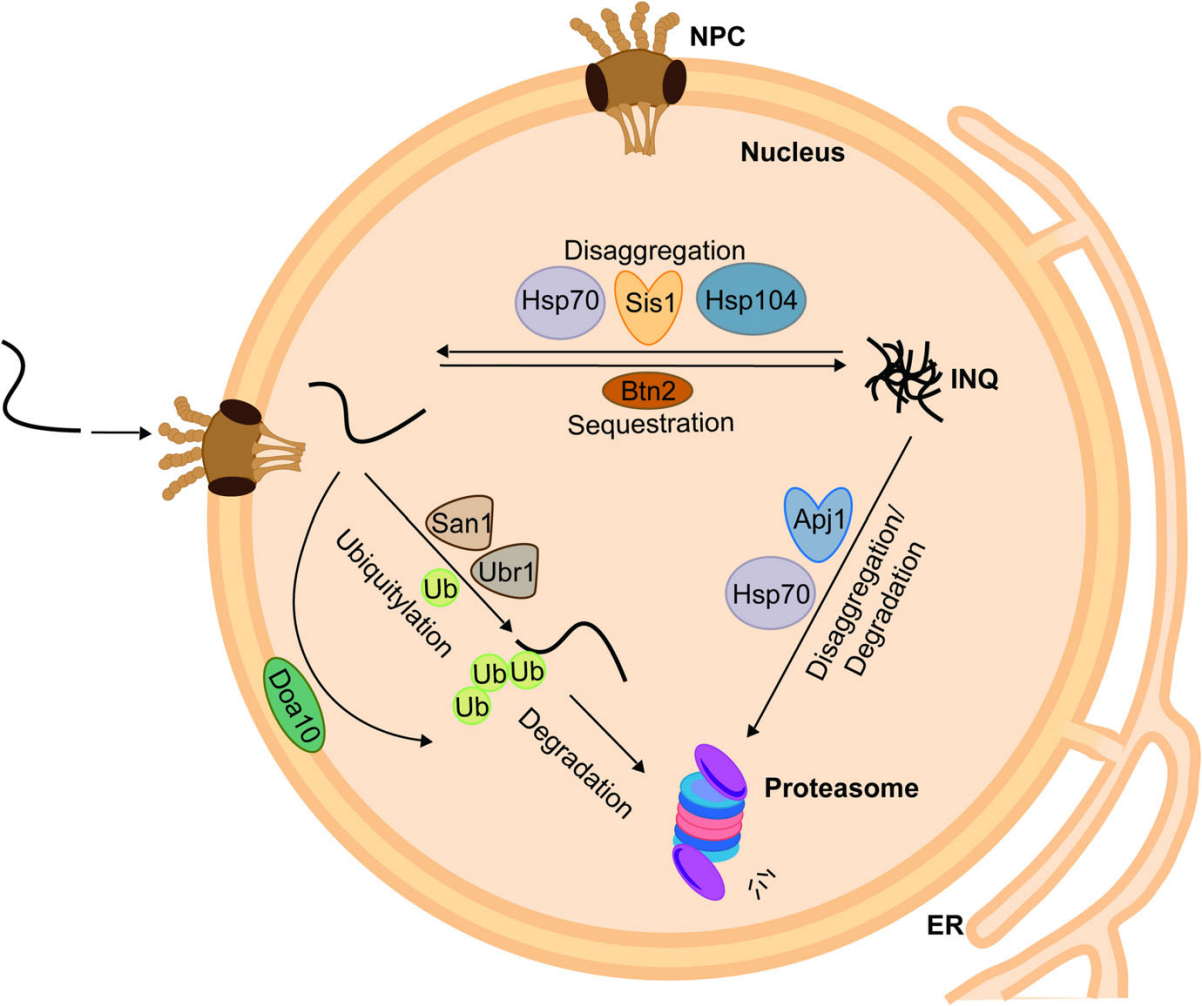
Nuclear protein quality control network. Proteins can be targeted to the nucleus, where they are subjected to diverse PQC strategies: (i) degradation via 26S proteasomes, (ii) sequestration into inclusions, and (iii) solubilization from inclusions by disaggregation. Central PQC components involved in the respective activities are indicated, including the E3 ligases San1, Ubr1, and Doa10, which mediate mitoPRE ubiquitylation and targeting to proteasomal degradation. The nuclear sequestrase Btn2 might deposit mitoPRE at the INQ inclusion. The J‐domain proteins Ydj1 and Sis1 and their partnering Hsp70 chaperones and the AAA+ disaggregase Hsp104 dissociate mitoPREs from INQ (disaggregation). Alternatively, this activity is executed by Apj1/Hsp70, presumably by targeting the disaggregated mitoPREs for degradation.

To avoid inappropriate interactions with other proteins and to relieve the burden on PQC capacity, mitoPREs can also be sequestered into aggregates (Krämer et al., 2023). This alternative strategy becomes particularly relevant when proteasomal degradation of mitoPREs is restricted. Yeast cells form inclusions containing mis‐ or unfolded proteins through the action of small heat shock proteins (sHsps) (Miller et al., 2015). sHsps bind to misfolded proteins to form sHsp/substrate complexes of variable sizes, thereby preventing unspecific aggregation (Reinle et al., 2022). The yeast sHsp Hsp42 sequesters misfolded proteins into large inclusions termed the cytosolic quality control compartment (CytoQ, also termed Q‐bodies). In the nucleus, the sHsp‐like chaperone Btn2 triggers formation of the intranuclear quality control compartment (INQ) (Miller et al., 2015). Proteins can be recovered from CytoQ and INQ through the action of the disaggregase Hsp104 and its partnering Hsp70s Ssa1‐4 (Ho et al., 2019; Miller et al., 2015). Alternatively, CytoQs can be degraded by ubiquitin‐dependent autophagy utilizing the autophagy receptor Cue5 (Marshall et al., 2016). Sequestration of mitoPREs via Hsp42 into a specialized form of CytoQ termed MitoStores was recently described, which are present on the mitochondrial surface (Figure 2) (Krämer et al., 2023). Formation of MitoStores is triggered in cells with reduced proteasomal degradation upon inhibition of mitochondrial import, in line with a role in relieving the proteostasis network under such conditions. The temporary sequestration into MitoStores provides the opportunity to rescue mitoPREs in an Hsp104‐dependent manner after the stress is relieved and resume their targeting to mitochondria (Figure 2).

## 4 NUCLEAR PQC IN *SACCHAROMYCES CEREVISIAE*

Over the past decade, the complexity of the nucleus as a hub for protein quality control (PQC) has been intensively studied. Like the cytosol and other cellular compartments, the nucleus has to deal with the burden of protein misfolding and subsequent formation of aggregates to maintain its proteome.

Where does this burden for the nuclear PQC mainly come from? A first source naturally comes from the proteins residing in the nucleus. For instance, proteins functioning in chromatin remodeling, genome integrity and splicing form inclusions inside the nucleus upon genotoxic stress such as MMS, UV radiation and heat stress (Gallina et al., 2015; Mathew et al., 2017; Tkach et al., 2012). Moreover, work done in the Pincus lab has shown that aggregation‐prone orphan ribosomal proteins accumulate around the nucleolus following heat stress and function as potent activators of the heat shock response (HSR) (Ali et al., 2023). Emerging evidence suggests that in *Saccharomyces cerevisiae* the nucleus functions as a general hub in protein quality control, as it was found to handle misfolded proteins from other compartments as well (den Brave et al., 2020; Park et al., 2013; Prasad et al., 2010; Shakya et al., 2021). In line with this, upon cellular stress PQC factors re‐localize to the nucleus to safeguard overall proteostasis (Kohler et al., 2024). These findings qualify the nucleus as a central PQC compartment.

A main nuclear PQC activity involves degradation of misfolded proteins (Figure 2). The 26S proteasome complex localizes to the yeast nucleus under different conditions at high concentrations (Russell et al., 1999), making it the central and highly efficient system for protein turnover inside the nuclear compartment. Several nuclear E3 ubiquitin ligases have been identified to promote the ubiquitin‐mediated degradation of misfolded proteins. San1 co‐operates with the E2 conjugating enzymes Cdc34 and Ubc1 to ubiquitylate its substrates (Gardner et al., 2005). San1 has a high avidity for its substrates due to the presence of several disordered regions, which act as redundant substrate binding sites (Ibarra et al., 2021; Rosenbaum et al., 2011). San1 does not require assistance by chaperones for substrate ubiquitylation. However, the yeast Hsp70 chaperones Ssa1/Ssa2 might help to maintain the solubility of San1 substrates and keep hydrophobic regions exposed for recognition by San1 (Fredrickson et al., 2011; Jones et al., 2020). Another nuclear‐residing E3 ligase is Ubr1, which additionally acts in the cytosol. Ubr1 has multiple activities, as it recognizes misfolded proteins but also folded substrates harboring destabilizing N‐termini (N‐end rule pathway) or internal degrons (Bartel et al., 1990; Eisele & Wolf, 2008; Khosrow‐Khavar et al., 2012; Mogk et al., 2007). Ubr1 activity towards misfolded proteins can be dependent on Hsp70 in vivo; however, in vitro, Ubr1 exhibits autonomous ubiquitylation activity towards, for example, unfolded Luciferase (Peters et al., 2025). Hsp70 might again keep substrates soluble and accessible for Ubr1 activity, though a direct handover of substrates from Hsp70 to Ubr1 has also been suggested (Singh et al., 2020). Ubr1 and San1 have overlapping functions in nuclear PQC, and substrate stabilization typically requires loss of both E3 ligases (Prasad et al., 2018).

The third identified E3 ligase acting in nuclear PQC is Doa10, which is enriched in the inner nuclear envelope and also in the ER membrane (Prasad et al., 2018). Doa10 recognizes substrates with a C‐terminal motif comprised of an amphipathic helix exposing a hydrophobic surface and an unstructured hydrophobic C‐terminal tail (Furth et al., 2011).

Refolding of substrates represents another branch of nuclear PQC and mainly relies on Hsp70. In the nuclear compartment, Hsp70 together with its partner JDP Sis1 is required to prevent aggregation of orphan ribosomal proteins during heat shock and to keep them in a liquid‐like state for efficient recycling during a recovery period (Ali et al., 2023). Indeed, upon Sis1 depletion or Hsp70 inhibition, the orphan ribosomal protein condensates become aberrantly solid, which negatively impacts cellular fitness (Ali et al., 2023).

When degradation and refolding capacities of nuclear PQC become overwhelmed upon stress conditions misfolded proteins accumulate. Similar to the sequestration of proteins into inclusions in the cytosol, intranuclear inclusions (INQ: intranuclear quality control compartment) can be formed, which are located adjacent to the nucleolus (Figure 2) (Gallina et al., 2015; Miller et al., 2015). INQ formation is induced upon DNA damaging conditions like MMS or UV radiation (Gallina et al., 2015) and following heat stress, also in combination with proteasome inhibition through MG132 (den Brave et al., 2020; Mathew et al., 2017). INQ formation requires the nuclear sequestrase Btn2 (Miller et al., 2015). Btn2, originally described as a v‐SNARE‐binding protein, shares some features with the classical sHsps like an α‐crystallin‐like core domain flanked by a disordered NTD and CTD (Ho et al., 2019).

Recovery of proteins from INQ is mediated by the Hsp104 disaggregase in conjunction with Sis1/Hsp70 (Figure 2) (Ho et al., 2019). Indeed, loss of Hsp104 and Sse1, an Hsp70 co‐chaperone, leads to a more pronounced accumulation of intranuclear inclusions during stress (Mathew et al., 2017). The sequestrase Btn2 plays an active role in substrate recovery by recruiting the JDP Sis1, which enables targeting of Hsp70, which subsequently recruits Hsp104 (Ho et al., 2019). Besides refolding of substrates deposited at INQ, an alternative pathway that can handle intranuclear inclusions has been described recently (den Brave et al., 2020). This pathway involves the nuclear JDP Apj1, which likely arose from a duplication event of the main cytosolic JDP Ydj1 (Sahi et al., 2013). Apj1 together with Hsp70 but independently of Hsp104 extracts substrates from INQ and primes them for proteasomal degradation (Figure 2) (den Brave et al., 2020). A role of Apj1 in the degradation of substrates is further confirmed by the strong negative genetic interaction with the nuclear SUMO‐targeted ubiquitin ligase Slx5, as deletion of both genes leads to the substantial accumulation of SUMOylated proteins and a temperature‐sensitive growth phenotype (Sahi et al., 2013). How processing of substrates at INQ by either Sis1/Hsp70/Hsp104 and Apj1/Hsp70 is orchestrated is unknown, but full INQ stabilization was only seen upon loss of both disaggregation systems (den Brave et al., 2020), indicating that they work in parallel.

It is worth noting that the abovementioned branches of the nuclear PQC are not isolated from one another: for instance, deletion of the sequestrase Btn2 in a *fes1Δ hsp104Δ* background, which suffers from low Hsp70 capacity, worsens the growth phenotype of yeast cells and induces the expression of the proteasome components (Jawed et al., 2022). However, the increased proteasomal activity is deleterious to these cells. Linking the deletion of *RPN4*, a transcription factor controlling proteasome expression, to the *fes1Δ hsp104Δ btn2Δ* cells can ameliorate the observed growth defects and attenuate stress responses (Jawed et al., 2022). This indicates that refolding and degrading PQC activities must be balanced and showcases that the branches of the PQC system must be highly coordinated to ensure proteome functionality.

## 5 HANDLING OF mitoPREs BY NUCLEAR PQC

A systematic analysis of GFP tagged mitoPREs destiny upon import inhibition via FCCP treatment revealed that 6.4% of these precursors are sorted to the nucleus for PQC, while for a large fraction the localization was unclear presumably due to rapid degradation (42.2%) (Shakya et al., 2021). These imported mitoPREs include matrix enzymes like components of the TCA cycle. Additionally, mitochondrial ribosomal proteins and carrier proteins from the inner mitochondrial membrane are targeted to the nucleus (den Brave et al., 2020). Notably, carrier proteins are not components of MitoStores, pointing to a specific role of the nuclear PQC in handling this class of mitoPREs (Krämer et al., 2023). The cellular machinery targeting mitoPREs to the nucleus and the specific sequence or structural determinants of mitoPREs recognized are currently unknown.

The primary strategy of the nuclear PQC system in handling non‐imported mitoPREs is similar to those applied by cytosolic PQC: mitoPREs are predominantly targeted to proteasomal degradation, a pathway termed mitoNUC (Figure 2) (Shakya et al., 2021). This underlines that non‐imported precursors cannot fold properly outside of mitochondria due to the absence of interaction partners or interference of the non‐cleaved MTS with their folding processes. Additionally, mitoPREs might pose a threat to cytosolic and nuclear protein homeostasis by undergoing aberrant interactions. Indeed, mitochondrial ribosomal proteins can interact with cytosolic ribosomal components during ribosome biogenesis inside the nucleus (Flohr et al., 2025), rationalizing mitoPRE degradation as the main nuclear PQC activity.

The E3 ligases San1, Ubr1 and Doa10 represent the core components of mitoNUC and mediate the ubiquitylation of mitoPREs (Figure 2). Full stabilization of imported mitoPREs is only observed in respective triple knockout cells, indicating overlapping and compensatory activities (Shakya et al., 2021). The same E3 ligases are also responsible for the turnover of non‐mitochondrial unfolded proteins, indicating that mitoPREs are subjected to ordinary nuclear PQC (Heck et al., 2010; Hickey et al., 2021; Prasad et al., 2010). Preventing nuclear targeting of a mitoPRE by fusion of a nuclear export sequence (NES) protected it from proteasomal degradation and created toxicity (Shakya et al., 2021). This rationalizes the nuclear targeting of specific mitoPREs and suggests their faster and more efficient degradation inside the nucleus. This increased proteolytic efficiency can be explained by high local concentrations of E3 ligases and 26S proteasomes (Russell et al., 1999).

Unexpectedly, the functional relevance of mitoNUC is currently unclear as *san1Δ ubr1Δ doa10Δ* knockout cells do not exhibit a growth phenotype and are not more sensitive to the uncoupler FCCP as compared to yeast wild‐type cells (Shakya et al., 2021). This points to the existence of alternative nuclear PQC pathways acting in parallel to protein degradation and functioning as a backup system. Indeed, stabilization of mitoPREs in E3 ligase mutants or by proteasome inhibition triggered their sequestration into nuclear inclusions (Figure 2) (den Brave et al., 2020; Shakya et al., 2021). This consequence is reminiscent of mitoPRE targeting to MitoStores in *rpn4Δ* cells (Krämer et al., 2023), further documenting common strategies of cytosolic and nuclear PQC. mitoPRE inclusions likely represent the intranuclear quality control compartment (INQ). Accordingly, co‐localization of Qcr6, a component of the respiratory chain complex III, with INQ was reported previously (Gallina et al., 2015). Notably, the MTS of mitoPREs is dispensable for nuclear import, yet it is essential for their degradation and sequestration and therefore might serve as direct recognition determinant for E3 ligases and sequestrases (Shakya et al., 2021). The sequestration of mitoPREs at INQ will keep them in an inert state and mitigate their potential toxicity. Notably, it is currently unclear whether the nuclear sequestrase Btn2, which is essential for INQ formation during, for example, heat stress (Miller et al., 2015), is also crucial for mitoPRE deposition or whether other/additional factors are involved. Thus, the relevance of mitoPRE sequestration as backup activity to proteasomal degradation has not been evaluated so far.

An additional nuclear PQC pathway of mitoPREs involves the nuclear JDP Apj1 (den Brave et al., 2020). Stabilizing Apj1 substrate interaction in a “HPD” trap mutant deficient in Hsp70 cooperation revealed binding to mitochondrial carrier and ribosomal proteins, and binding was strongly increased upon MG132‐mediated inhibition of 26S proteasomes (den Brave et al., 2020). The function of Apj1 is so far linked to proteasomal degradation of bound substrates. The disaggregation activity of the Apj1/Hsp70 system might ensure that mitoPREs deposited at INQ are ultimately targeted to proteasomal destruction (Figure 2). How exactly Apj1/Hsp70 activity is coordinated and intertwined with the other nuclear PQC pathways in mitoPRE handling remains to be explored.

## 6 SIGNALING OF MITOCHONDRIAL IMPORT STRESS TO STRESS RESPONSE PATHWAYS

**FIGURE 3:**
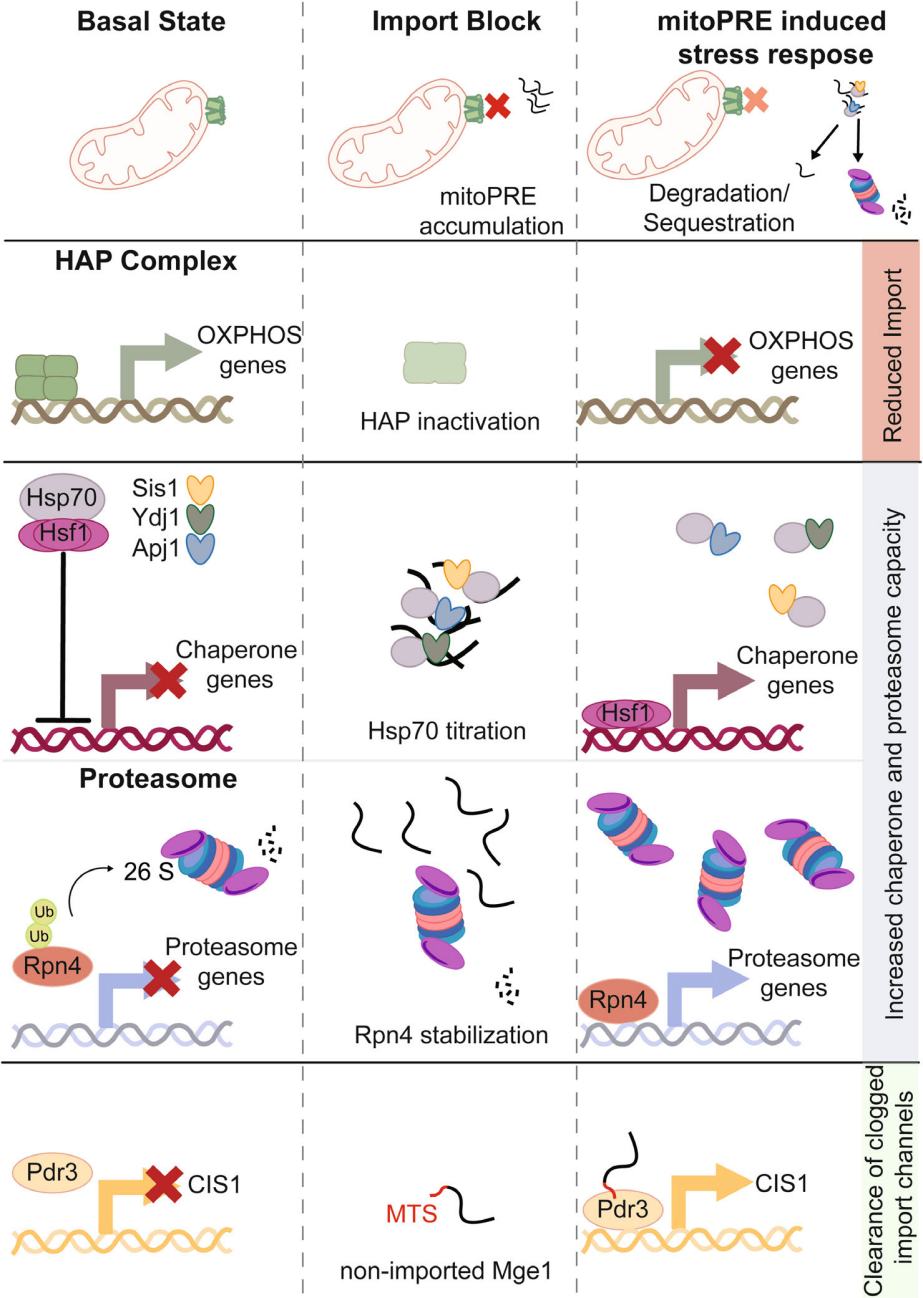
mitoPRE‐induced stress responses. Accumulation of mitoPREs upon import block triggers diverse stress response pathways. Inactivation of the HAP complex reduces expression of OXPHOS genes, encoding for components of oxidative phosphorylation in the inner mitochondrial membrane. This reduces the burden for the limited import capacity. Binding of mitoPREs to Hsp70 releases Hsf1 from Hsp70 inhibition, triggering increased expression of chaperone genes. Degradation of mitoPREs by 26S proteasome leads to stabilization of Rpn4, leading to enhanced expression of genes encoding for proteasome subunits. Increased levels of chaperones and 26S proteasomes allow for efficient handling of mitoPREs. Non‐imported Mge1 binds via its mitochondrial targeting sequence (MTS) to Pdr3 in the nucleus, triggering expression of *CIS1*. Cis1 targets the AAA+ protein Msp1 to the clogged import channel for clearance from arrested mitoPREs.

Mitochondrial import defects caused by, for example, expression of a clogger construct trigger compensatory transcriptional responses that act at diverse levels. These adaptive expression changes within the nucleus include (i) downregulation of mitochondrial gene expression, increased expression of (ii) PQC components and (iii) of factors that clear blocked mitochondrial import channels (Figure 3) (Boos et al., 2020; Pfanner et al., 2025).

In yeast, import inhibition causes inactivation of the HAP complex leading to downregulation of components of oxidative phosphorylation (OXPHOS), thus reducing the burden for the limited import capacity (Boos et al., 2019). To adjust the capacity of the cytosolic and nuclear PQC systems to the accumulation of mitoPREs, the transcription factors Rpn4 and Hsf1 become activated to trigger enhanced expression of components of the UPS system (via Rpn4) and of molecular chaperones (via Hsf1) (Boos et al., 2019). Through these transcriptional responses, enhanced degradation but also sequestration and recovery of mitoPREs are ensured. Finally, activation of the transcription factor Pdr3 increases expression of factors that liberate clogged import channels (Weidberg & Amon, 2018).

Rpn4 is an unstable transcription factor and targeted to proteasomal degradation in non‐stressed cells (Dohmen et al., 2007). An overload of proteasomal capacity by enhanced generation of substrates thus triggers Rpn4 stabilization. Increased Rpn4 levels in turn enhance expression of proteasome subunits and assembly factors, adjusting proteasomal capacity to the actual workload and initiating a negative feedback loop to limit Rpn4 activity. The accumulation of mitoPREs upon import block operates in the same way, stabilizing Rpn4 and triggering the Rpn4‐dependent UPRam (unfolded protein response to mistargeting of mitochondrial proteins) (Figure 3) (Boos et al., 2019; Wrobel et al., 2015).

The activity of Hsf1 is primarily controlled by Hsp70 chaperones, which inactivate Hsf1 through direct binding (Masser et al., 2019). Titration of Hsp70 chaperones via accumulation of misfolded protein substrates upon, for example, heat shock liberates Hsf1 to trigger enhanced expression of chaperones including Hsp70 (Figure 3) (Krakowiak et al., 2018; Zheng et al., 2016). Refolding of misfolded protein species and increased Hsp70 levels ultimately lead to the attenuation of the HSR. Various partnering JDPs are implicated in targeting Hsp70 to Hsf1, including Sis1 and Ydj1 (Ali et al., 2023; Feder et al., 2021; Ruger‐Herreros et al., 2025). The nuclear JDP Apj1 has been specifically linked to the attenuation phase of the HSR by displacing Hsf1 from heat shock promoters (Ruger‐Herreros et al., 2025). Its central role in nuclear PQC allows Apj1 to directly link the presence of misfolded and aggregated proteins inside the nucleus to Hsf1 activity control. Repair or removal of the damaged proteins will liberate Apj1 to target DNA‐bound Hsf1 and to shut off the stress response. How can mitoPREs trigger Hsf1 activation? Hsp70s and its JDP co‐chaperones Sis1 and Ydj1 are involved in the post‐translational import of mitoPREs (Jores et al., 2018), and mitoPRE accumulation will thus titrate Hsp70 and JDPs (Drwesh et al., 2022). Furthermore, the nuclear JDP Apj1 also interacts with mitoPREs (den Brave et al., 2020) and could function as a mediator by directly adjusting Hsf1 activity to the status of mitochondrial protein import. Regardless of the JDP involved, mitoPRE accumulation will reduce Hsp70 capacity and thus liberate Hsf1 from Hsp70 inhibition (Figure 3).

Restoring the functionality of clogged mitochondrial import channels is the final response triggered upon import stress. MitoCPR (mitochondrial compromised protein import response) is driven by the transcription factor Pdr3, which controls expression of, for example, *CIS1* (Weidberg & Amon, 2018). Cis1 represents an adaptor protein, which recruits the AAA+ unfoldase Msp1 to blocked TOM complexes, mediating the removal of stalled precursors, liberating the clogged channel. It was recently demonstrated that non‐imported Mge1, which inside mitochondria functions as co‐chaperone of mitochondrial Hsp70, is targeted to the nucleus to bind and activate Pdr3 (Figure 3) (Yuan et al., 2026). The MTS of Mge1 is essential and sufficient for Pdr3 activation, documenting a novel role of an MTS as a stress signaling molecule.

The individual stress responses are intertwined as Hsf1 controls *RPN4* expression, while *PDR3* represents a target gene of Rpn4. This Hsf1‐Rpn4‐Pdr3 transcriptional cascade supports a coordinated stress response, which can simultaneously preserve cellular proteostasis and restore mitochondrial import capacity (Boos et al., 2020). Each branch of this network still requires distinct activation through mitoPRE accumulation, ensuring that only import defects trigger such stress responses.

## 7 FINAL REMARKS

Nuclear targeting represents a novel strategy of dealing with non‐imported mitoPREs. Here, the nucleus functions as a compartment for degradation or sequestration of mitoPREs, which may serve as a strategy to avoid interference of these proteins with protein synthesis at ribosomes in the cytosol. In fact, PQC of non‐nuclear proteins in the nucleus appears to be a general strategy conserved from yeast to human (Park et al., 2013). However, whether mitoPREs are subjected to nuclear PQC in human cells is currently unknown. Some components of yeast nuclear PQC are conserved in human (e.g., E3 ligases Ubr1: UBR1, Doa10: MARCH6/TEB4) while others are not (e.g., San1, Btn2, Apj1). Therefore it remains to be determined whether mitoPREs are subjected to the same PQC activities in nuclei of human cells.

The same PQC activities as in the yeast nucleus operate in the cytosol, raising the question why some mitoPREs are specifically routed to the nucleus. Proteasomal degradation of at least some mitoPREs is more efficient in the nucleus, presumably due to the high local abundance of proteasomes (Shakya et al., 2021), providing an initial rationale. Interestingly, nuclear targeting may also provide a mechanism to link impaired mitochondrial import to specific compensatory stress responses as it has been exemplified for non‐imported Mge1 (Yuan et al., 2026). A similar mechanism has been described in *Caenorhabditis elegans* for the matrix targeted protein ATFS‐1, which translocates to the nucleus and induces a transcriptional program when not imported into mitochondria (Nargund et al., 2012). Whether other nuclear targeted mitoPREs can have similar signaling roles, for example through interaction with the JDP Apj1, which regulates the HSR, remains to be elucidated (den Brave et al., 2020; Ruger‐Herreros et al., 2025). Furthermore, it is not clear how mitoPREs are sorted to the nucleus and what the determinants within mitoPREs for nuclear import are. Addressing these questions will help to understand the complex and central role of the nucleus in mitochondrial protein biogenesis.

## AUTHOR CONTRIBUTIONS

**Axel Mogk:** Writing – original draft; writing – review and editing; conceptualization. **Stefano Rossi:** Writing – review and editing; writing – original draft. **Kira Ritzenhofen:** Writing – review and editing; visualization; writing – original draft. **Fabian den Brave:** Writing – original draft; writing – review and editing; conceptualization.

## FUNDING INFORMATION

This work was supported by grants of the Deutsche Forschungsgemeinschaft to F.d.B. (BR 6283/5‐1, project ID 529716110; SPP 2453 BR 6283/6‐1, project ID 541596792) and to A.M. (SPP2453, MO970/9‐1, project ID 541596792).

## CONFLICT OF INTEREST STATEMENT

The authors declare that they have no competing financial or non‐financial interests related to the work.

## Data Availability

Data sharing not applicable to this article as no datasets were generated or analysed during the current study.
